# Antofine suppresses endotoxin‐induced inflammation and metabolic disorder via AMP‐activated protein kinase

**DOI:** 10.1002/prp2.337

**Published:** 2017-07-25

**Authors:** Shao‐Ting Chou, Fang Jung, Shih‐Hsing Yang, Hwei‐Ling Chou, Guey‐Mei Jow, Jau‐Chen Lin

**Affiliations:** ^1^ Division of Chest Medicine Department of Internal Medicine Kaohsiung Armed Forces General Hospital Kaohsiung Taiwan; ^2^ Department of Respiratory Therapy Fu‐Jen Catholic University New Taipei City Taiwan; ^3^ School of Medicine Fu‐Jen Catholic University New Taipei City Taiwan; ^4^ Medical Department Kaohsiung Armed Forces General Hospital Kaohsiung Taiwan

**Keywords:** AMPK, Antofine, endotoxin, inflammation, metabolism

## Abstract

The inhibition of activated macrophages has been used to develop anti‐inflammatory agents for therapeutic intervention to human diseases that cause excessive inflammatory responses. Antofine, a phenanthroindolizidine alkaloid, has a potent anti‐inflammatory effect. However, the molecular mechanisms of its anti‐inflammatory activity have not yet been fully detailed. In this study, we comprehensively explored the anti‐inflammatory effects of antofine on endotoxin‐induced inflammation in macrophages using cDNA microarray analysis, thereby elucidating the potential mechanism by which antofine suppresses inflammation. Antofine significantly suppressed the secretion of proinflammatory cytokines such as TNF *α* and IL‐1*β* and the production of iNOS in LPS‐activated Raw264.7 macrophage cells. In addition, antofine can suppress the expressions of several inflammation‐related genes (such as ARG‐1, IL1F9, IL‐10, and IL‐33) and extracellular matrix genes (such as TNC and HYAL1), as well as a vasopressor gene (EDN1) in activated macrophage cells, that are induced by LPS stimulation. The gene expression profiles analyzed by GeneMANIA software showed that antofine not only contributed anti‐inflammatory activity but also modulated the cellular metabolism via AMPK. Furthermore, antofine also modulated the activation of AMPK and caspase‐1, the key regulator in inflammasome‐mediated IL‐1*β* maturation, in activated macrophage cells. In conclusion, these data indicated that antofine potentially can not only contribute an anti‐inflammatory effect but can also attenuate the metabolic disorders induced by inflammation via AMPK.

**Abbreviations**
AMPK: AMP‐activated protein kinase
ARG‐1: arginase 1
EDN1: Endothelin 1
HYAL1: Hyaluronoglucosaminidase 1
IL‐10: interleukin 10
IL1F9: interleukin‐1 family member 9
IL‐1: interleukin‐1
IL‐33: interleukin 33
iNOS: inducible nitric oxide synthase
LPL: lipoprotein lipase
LPS: Lipopolysaccharide
NF‐kB: nuclear factor kappa‐light‐chain‐enhancer of activated B cells
PCR: polymerase chain reaction
PGEs: prostaglandins
ROS: reactive oxygen species
SIRS: systemic inflammatory response syndrome
TNC: Tenascin C
TNF*α*: tumor necrosis factor‐*α*

## Introduction

Endotoxin‐induced acute and excessive inflammatory responses in patients including endotoxemia and systemic inflammatory response syndrome (SIRS) still cause high mortality despite advanced supportive care and clinical therapeutic intervention (Cohen 2002). Lipopolysaccharide (LPS), a bacterial endotoxin, can stimulate the acute release of cytokines such as tumor necrosis factor‐*α* (TNF*α*) to mediate the damage caused by inflammation (Tracey et al. 1986, 1987). TNF*α* can enlarge and extend the inflammatory response by stimulating cells to secrete other cytokines including interleukin‐1 (IL‐1) and mediators such as prostaglandins (PGEs), nitric oxide, and reactive oxygen species (ROS), which amplify inflammation and cause tissue injury (Bradley 2008; Dinarello 2011). The immune cells, including macrophages and neutrophils, are thought to play a critical role in human immune responses to bacterial infections during the process of endotoxemia. Relatedly, the inhibition of activated macrophages has been used to develop potential anti‐inflammatory compounds attenuating human diseases due to excessive inflammatory responses.

Antofine, a phenanthroindolizidine alkaloid, has previously been shown to exhibit antiviral (Gao et al. 2012; Wang et al. 2012) and antitumorigenic activities (Staerk et al. 2002; Fu et al. 2007), including suppressive effects on pancreatic cancer cells, by inhibiting the activation of NF‐kB (Shiah et al. 2006). Recently, it has also been reported that antofine can also exert potent anti‐inflammatory effects, by suppressing the production of nitrite oxide induced by LPS challenge in murine macrophage cells (Min et al. 2010), and antiadipogenic effects via the direct suppression of PPAR*γ* protein expression in adipocyte cells (Jang et al. 2014). However, the details of the mechanism by which antofine exerts these anti‐inflammatory and antiadipogenic effects remain unclear.

AMP‐activated protein kinase (AMPK) is an important regulator of whole‐body energy metabolism that mediates energy homeostasis including carbohydrate, lipid, and protein metabolism. However, dysregulation of AMPK causes obesity, metabolic syndrome, cardiovascular disease, and cancer (Steinberg and Kemp 2009). Several studies have shown that reduced AMPK activity is correlated with inflammation in adipose tissue and macrophages (Yang et al. 2010; Gauthier et al. 2011). The activation of AMPK activity by AICAR, an activator of AMPK, can inhibit LPS‐induced inflammatory responses in an in vitro model, as well as the inflammation resulting from cystic fibrosis and lung injury in several animal models (Zhao et al. 2008; Myerburg et al. 2010). In this study, we comprehensively explored the anti‐inflammatory effect of antofine on LPS‐induced inflammation in macrophages using cDNA microarray analysis. We found that antofine not only contributes an anti‐inflammatory effect but also an antifibrogenic effect, which together cause the suppression of the formation of extracellular matrix. In addition, this is the first study to identify the crosstalk between antofine and the activation of AMPK activity, which can suppress the inflammatory response in macrophages.

## Materials and Methods

### Cell culture

The cells including Raw264.7 murine macrophage and human bronchial epithelial (BEAS‐2B) both were gotten from BCRC (Bioresource Collection and Research Center, Hsinchu, Taiwan). The cells were grown in normal DMEM (Hyclone, Logan, UT) supplemented with 1.5 g/L of NaHCO3, 4.5 g/L glucose, as well as 10% fetal bovine serum (FBS, Hyclone), MEM nonessential amino acid (Hyclone), 100 mM sodium pyruvate (Hyclone), and antibiotics (Hyclone).

### Chemicals

**Figure 1:**
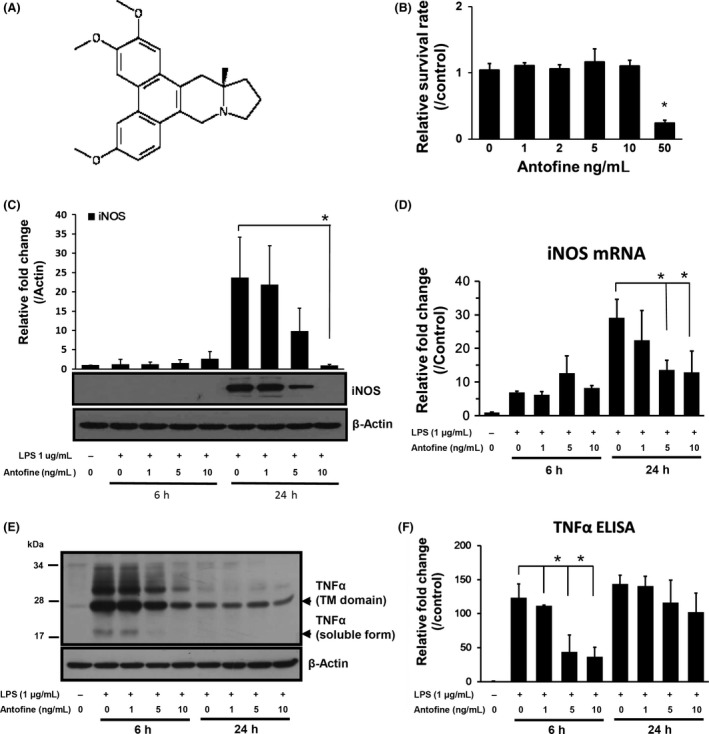
Antofine suppressed the production iNOS and the secretion of proinflammatory cytokines. (A) The chemical structure of antofine. (B) Cell viability of Raw264.7 macrophages exposed to various concentrations of antofine for 48 h was measured by WST‐1 assay. (C) Macrophage cells were pretreated with various concentrations of antofine for 30 min and then stimulated by LPS (1 *μ*g/mL) for 6 h and 24 h. The production of iNOS in treated cells was measured by immunoblot. The densitometry of specific bands on the blot was measured by Image J software (NCBI). The expression of *β*‐actin served as an internal control for protein amount loading. (D) The expression of mRNA of the iNOS gene was quantified by quantitative real‐time PCR. The expression of *β*‐actin served as an internal control for RNA quantity. (E) The production of TNF *α* in treated cells was measured by immunoblot. The 26 kDa fragment protein as indicated was the transmembrane protein digested from pro‐TNF *α* and the 18 kDa fragment protein as indicated was the soluble form of TNF *α*. (F) the soluble form of TNF *α* in cultured medium was measured by ELISA method. The experiments were performed at least three times. *P <* *0.05 versus LPS alone stimulation.

The tested antofine (Fig. 1A, PubCnem CID 639288) was produced by Dr. KH Lee's laboratory (Dong et al. 1999; Liu et al. 1999). LPS (*Escherichia Coli* 0111:B4) was purchased from Sigma‐Aldrich (Saint Louis, MO).

### cDNA microarray

Total RNA was extracted and isolated from the cells incubated with/without the lipopolysaccharide (1 *μ*g/mL LPS; Escherichia Coli 0111:B4) alone or incubated with LPS plus antofine (10 ng/mL) for 24 h using TRIzol (Invitrogen). Five microgram of total RNA from each sample was performed in each cDNA microarray. Antisense RNA (aRNA) target was labeled using Amino Allyl MessageAmp II aRNA Amplification Kit (Ambion Inc., Aus‐ tin, TX). Cy5‐labeled RNA targets were hybridized to Mouse Whole Genome OneArray v2 (Phalanx Biotech Group), and the signal of hybridized spots in chip were detected by the Axon 4000 scanner (Molecular Devices). The intensity of each spot was analyzed by Genepix 4.1 software (Molecular Devices) (Lin et al. 2009; Morales et al. 2014). The microarray experiments were adhered to the guidelines of the Microarray Gene Expression Data Society (www.mged.org/Workgroups/MIAME/miame_checklist.html).

### Real‐time reverse transcriptase polymerase chain reaction

Total RNA was extracted from the cultured cells using TRIzol reagent (Invitrogen), and then quantified using an Epoch Microplate Spectrophotometer with a Take3 Micro‐Volume Plate (BioTek, Winooski, VT). One microgram of total RNA was reverse‐transcribed using an MMLV reverse transcriptase kit with random primers (Epicentre Biotechnologies, Madison, WI). The specific primers for real‐time quantitative PCR were designed by qPrimerDepot (http://primerdepot.nci.nih.gov/) (Table 1). The real‐time PCR reactions were performed in 20 *μ*L volumes containing 10 *μ*L of Real‐time PCR DyNAmo Flash SYBR Green qPCR reagent (Thermo Fisher Scientific Inc., NYSE: TMO). The PCR reaction was performed using a PikoReal™ 96 Real‐Time PCR System (Thermo Fisher Scientific Inc.), and the conditions were as follows: 45 cycles of 95°C for 5 sec and 60°C for 1 min. The data were analyzed using PiKoReal Software 2.0, exported into Excel for analysis using the ΔC_t_ (the number of PCR cycles to reach the threshold of the product detection) method, and normalized to *β*‐actin as an internal control. The quantitative real‐time PCR experiments adhered to the MIQE (Minimum Information for Publication of Quantitative Real‐Time PCR Experiments) guidelines (http://www.clinchem.org/cgi/content/short/55/4/611).

**Table 1:** Designed primers for real‐time PCR**.**

Gene	Forward primer (5′)	Reverse primer (3′)	Size (bp)
IL1F9	GAACAGGACAAAGGGATTGC	AGGGTGGTGGTACAAATCCA	132
ARG1	GGAACCCAGAGAGAGCATGA	TTTTCCAGCAGACCAGCTTT	132
TNC	CATCTCAGGGCTTCCACCTA	TCTGGAGTGGCATCTGAAAC	147
IL10	TTTGAATTCCCTGGGTGAGA	AGACACCTTGGTCTTGGAGC	140
IFNB1	AATTTCTCCAGCACTGGGTG	AGTTGAGGACATCTCCCACG	135
CCR2	TGCCATCATAAAGGAGCCAT	TCCTTTGATTTGTTTTTGCAGAT	132
DDX4	ATTCTTCTGGAGCAAATGGAGA	ACCTCTGTTTCCAAAGCCCT	131
HMOX1	ACAACCAGTGAGTGGAGCCT	TCAAGGCCTCAGACAAATCC	148
ALDH1B1	CTGCTGAACTGACCGGAGA	AAGAGGTAGTCCTGCCCTGG	150
CTH	TGCTAAGGCCTTCCTCAAAA	CAGTGTGTCATTGATCCCGA	148
PECAM1	CCTCCAGGCTGAGGAAAACT	GGTGCTGAGACCTGCTTTTC	143
SRC	CGGCTGCAGATTGTCAATAA	GTACCACTCCTCAGCCTGGA	147
F7	TCCAGGGACCTCTAGGGACT	AGCACTGTTCCTCATTGCAC	141
IL7R	ACGATCACTCCTTCTGGTGC	GCATTTCACTCGTAAAAGAGCC	140
HYAL1	GACATGCTTGGGCTTACACA	CCAACTCGAGCAAAGTCAGG	142
LAPTM4B	CTCGGTTCTACTCCCACAGC	TGATACTGATTGGGATCTGCC	139
NPC1	CCGAACTGAGAGCTGTAGCC	TATCTCCAGTCGCAATTCCA	150
MYC	TGAAGGCTGGATTTCCTTTG	TTCTCTTCCTCGTCGCAGAT	140
EDN1	CCAAGGAGCTCCAGAAACAG	TGTCCATCAAGGAAGAACAGG	137
IL33	CCTCCCTGAGTACATACAATGACC	GTAGTAGCACCTGGTCTTGCTCTT	114
INOS	GAAGAAAACCCCTTGTGCTG	GTCGATGTCACATGCAGCTT	138
COX2	GATGTTTGCATTCTTTGCCC	GGCGCAGTTTATGTTGTCTG	149
TNFα	CGCTCTTCTGTCTACTGAACTT	ATGAGATAGCAAATCGGCTGAC	357
IL1*β*	CGCAGCAGCACATCAACAAGAGC	TGTCCTCATCCTGGAAGGTCCACG	111
*β*‐ACTIN	GATTACTGCTCTGGCTCCTAGC	GACTCATCGTACTCCTGCTTGC	147

The specific primers were designed by qPrimerDepot (http://primerdepot.nci.nih.gov/)

### Enzyme‐linked immunosorbent assay

The concentrations of TNF*α* and IL‐1*β* in cultured medium were examined by ELISA kit (eBioscience, San Diego, CA) according to the manufacturer's instructions. The tested plates were analyzed at 450 nmol/L using an Epoch Microplate Spectrophotometer. The concentrations of TNF*α* and IL‐1*β* were calculated from the standard curve.

### Western blot analysis

Equal amounts of soluble protein extracted from cells were separated by SDS–PAGE and then transferred to a PVDF membrane (HybondTM‐P, Amershan, Piscataway, NJ). The blots were probed with specific antibodies to iNOS (BD transduction Lab., San Jose, CA), EDN1, HYAL‐1, ARG‐1 (GeneTex, Inc**,** Irvine, CA), Caspase‐1 (Millipore, Billerica, MA), IL‐1*β* (Abnova, Taipei City, Taiwan), TNF*α* (R&D Systems, Inc., Minneapolis, MN), p‐A‐CoA, p‐AMPK*α*, and (Cell Signaling, Beverly, MA), and *β*‐actin (Sigma‐Aldrich). The signal of electrochemical luminescence in probed blots was detected using FUJI Medical X‐ray film (FUJI Corporation, Kofu, Yamanashi, Japan). The densitometry of specific bands on the blot was measured by Image J software (NCBI).

### Cell viability assay

Cells were seeded at 10^5^ cells/well in each 96‐well plates. The cells were treated with antofine (0–50 ng/mL) in 0.2 mL of culture medium for 48 h. 20 *μ*L of WST‐1 (Roche) was added to each well and incubated at 37°C for 2 h. The absorbance of the samples was measured at 450 nm wavelength using a spectrophotometer (BioTek Instruments, Inc.).

### Statistical methods

The quantitative data are expressed as means ± SD. Statistical significant of difference between groups was analyzed using the *t*‐test. *P* < 0.05 were considered to be significant.

## Results

A cell viability assay was performed to elucidate whether or not the suppressive effect on the production of inflammation‐related genes was due to cell death, and the results showed that antofine exhibited no cell cytotoxicity in Raw264.7 macrophage cells at doses below 10 ng/mL but dramatically suppressed cell proliferation at a dose of 50 ng/mL (Fig. 1B). To evaluate the potential mechanism of antofine in inhibiting inflammation, Raw264.7 macrophage cells cotreated with LPS and antofine were harvested at 6‐h and 24‐h time points, and the total protein and RNA extracted from cells were analyzed using Western blotting and real‐time PCR, respectively. The Western blotting results showed that the production of iNOS proteins was dramatically induced by LPS stimulation at 24 h but was decreased in a dose‐dependent manner as the dose of antofine compound was increased (Fig. 1C). The real‐time PCR results also confirmed that the suppressive effect of antofine on the expression of the iNOS gene was due to the modulation of gene transcription (Fig. 1D). The Western blots results showed that the antofine dose dependently inhibited the production of full length TNF*α* induced by LPS stimulation at the 6‐h time point. However, the production of TNF*α* was no change in LPS‐treated cells at the 24‐h time point because the cells have been desensitized to LPS stimulation (Fig. 1E). The ELISA results also showed that secretion of TNF*α* (soluble form) in LPS‐stimulated macrophage cells were dramatically suppressed at the 6‐h time point. The increase secretion of TNF*α* in culture medium at the 24‐h time point when compared with 6‐h time point was due to accumulation of soluble TNF*α* for 24 h (Fig. 1F).

**Figure 2:**
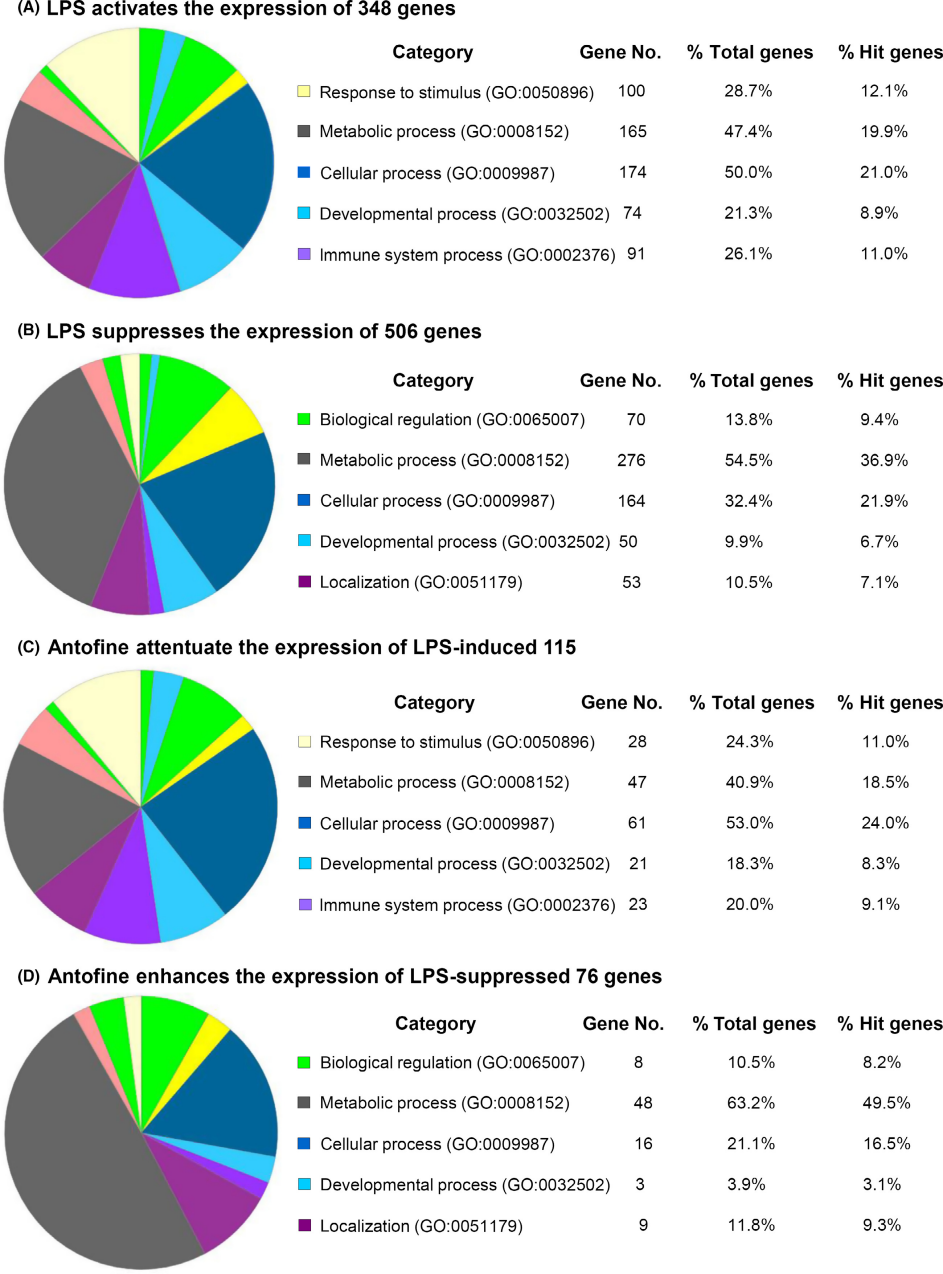
Gene expression profiles of Raw264.7 murine macrophage cells modulated by LPS and antofine. Raw264.7 macrophage cells were pretreated with or without antofine (10 ng/mL) for 30 min and then stimulated by LPS (1 *μ*g/mL) for 24 h. The mRNA of untreated, treated with LPS alone, and treated with LPS plus antofine cells were isolated and analyzed by cDNA microarray. The gene expression profiles were analyzed using software from the GeneMANIA and PANTHER web sites. In total, 854 putative genes showed a statistically significant fourfold difference in LPS stimulation cells compared with untreated control cells. (A) 348 of the 854 genes were upregulated by LPS stimulation. (B) 506 of the 854 genes were downregulated by LPS stimulation. (C) 115 of the 348 genes induced by LPS were suppressed by antofine treatment. (D) 76 of the 506 genes downregulated by LPS stimulation were enhanced by antofine treatment. These result indicated that antofine may reactivate metabolic pathways to suppress LPS‐induced inflammatory responses.

To elucidate the molecular mechanism by which antofine suppresses the LPS‐induced inflammation of cells, we used a cDNA microarray with free software tools from the Genemania and Panther web sites to analyze the changes in gene expression after cells were exposed to LPS (1 *μ*g/mL) with or without antofine (10 ng/mL). In total, 854 putative genes showed a statistically significant fourfold difference in expression in Raw264.7 cells after 24 h of LPS stimulation compared with untreated control cells. Of these 854 genes, 348 were upregulated and 506 were downregulated (Fig. 2A and B). The upregulated genes are those involved in responses to stimuli, as well as metabolic, cellular, development, and immune system processes. In contrast, biological regulation, metabolic process, and localization process genes were downregulated significantly by treatment with LPS. 115 of the 348 genes induced by LPS were suppressed by antofine treatment (Fig. 2C). Seventy‐six of the 506 genes downregulated by LPS were enhanced by antofine treatment (Fig. 2D). Interestingly, 48 (63.2%) of these 76 enhanced genes were involved in metabolic processes. This result indicated that antofine may reactivate metabolic pathways to suppress LPS‐induced inflammatory responses.

**Figure 3:**
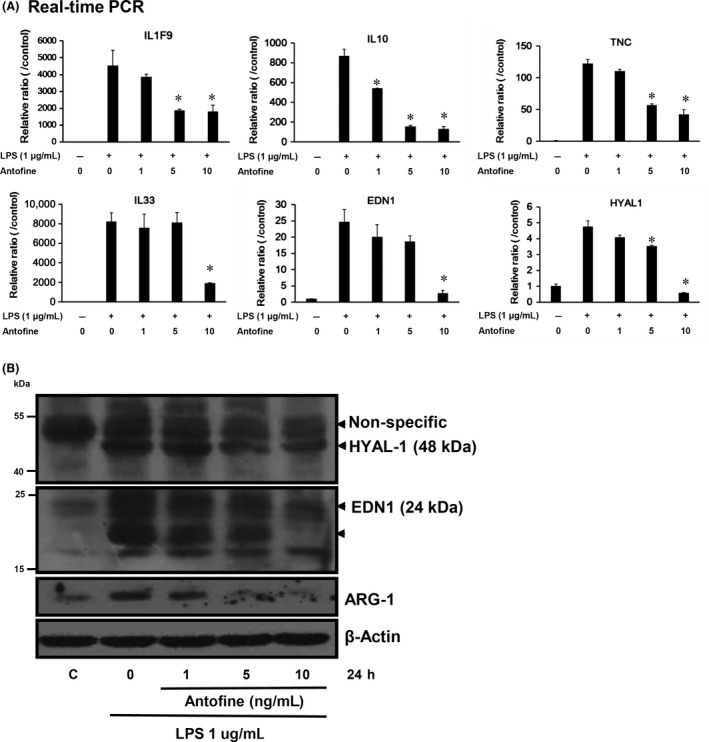
The expressions of inflammatory mediators and extracellular matrix genes were regulated by antofine. Raw264.7 macrophage cells were pretreated with antofine at the indicated doses ranging from 0 to 10 ng/mL for 30 min and then stimulated by LPS (1 *μ*g/mL) for 24 h. (A) Quantitative real‐time RT‐PCR was performed with specific primers including IL1F9, IL10, TNC, IL33, EDN1, and HYAL1 as shown in Table 1. The expression of *β*‐actin served as an internal control for RNA quantity. (B) The protein amount of these genes including HYAL1, EDN1, and ARG1 was analyzed by immunoblot. The expression of *β*‐actin served as an internal control for protein amount loading. *P <* *0.05 versus LPS alone stimulation.

In order to confirm the effects of antofine on the expression of inflammatory mediators, the mRNA or proteins of several inflammatory mediators were detected either by quantitative RT‐PCR or Western blotting, respectively. As shown in Table 2, the upregulated genes were enhanced by LPS at least threefold higher than they were in the untreated control cells (column 2), and they were suppressed by LPS plus antofine cotreatment, the effects of which were analyzed by cDNA array data (column 3). The fold changes in the expression levels of these genes (column 5, 6) in LPS alone or LPS plus antofine cotreated cells compared to control cells were detected and determined by real‐time PCR with specific designed primers (Table 1). To further verify the real‐time PCR results, Raw264.7 macrophage cells were pretreated with various concentrations of antofine for 30 min and then stimulated by LPS for 24 h, and the total protein and RNA extracted from the cotreated cells were analyzed using Western blotting and RT‐PCR, respectively. The results showed that the expressions of inflammatory cytokines (such as IL1F9, IL‐10, and IL‐33) and extracellular matrix genes (such as TNC and HYAL1), as well as that of a vasopressor gene (EDN1), were dose dependently downregulated by antofine treatment according the results of the real‐time PCR (Fig. 3A). The Western blots also confirmed that protein concentrations of LPS‐induced genes including HYAL‐1, EDN1, and ARG‐1 were dose dependently suppressed by antofine treatment (Fig. 3B).

**Table 2:** Affected genes related to inflammation

Symbol	cDNA Microarray, fold change (mean ± SD)		Molecular function	Real‐time PCR, fold change,(mean ± SD)	
	LPS alone /control	LPS + Antofine /control		LPS alone /control	LPS + Antofine /control
IL33	3375.1 ± 96.7	1620.4 ± 133.5	Cytokine	284878 ± 33433	77303 ± 26370
IL7R	42.8 ± 1.7	18.8 ± 1.8	Receptor activity	188.5 ± 35.8	38.7 ± 8.5
IL10	9.6 ± 1.9	3.0 ± 0.0	Cytokine	1258.2 ± 941.8	138.1 ± 1.4
ARG1	88.0 ± 0.5	7.8 ± 0.6	Hydrolase activity	1064.6 ± 411.9	101.3 ± 19.3
TNC	8.6 ± 0.6	2.4 ± 0.3	Receptor binding	233.5 ± 33.1	20.9 ± 5.9
ALDH1B1	6.8 ± 0.2	2.5 ± 0.1	Oxidoreductase activity	11.1 ± 3.9	3.1 ± 0.3
CTH	14.2 ± 1.1	3.1 ± 0.3	Lyase activity	12.4 ± 2.8	1.5 ± 0.4
MYC	17.3 ± 1.4	8.9 ± 0.5	Transcription factor	15.4 ± 4.3	4.0 ± 0.4
F7	3.0 ± 0.1	0.4 ± 0.2	Calcium ion binding	29.1 ± 1.7	3.3 ± 0.7
SRC	8.1 ± 0.4	3.7 ± 0.8	Tyrosine kinase activity	44.8 ± 1.8	29.1 ± 0.3
IFNB1	4.7 ± 0.5	1.6 ± 0.1	Cytokine receptor binding	26.7 ± 0.8	2.7 ± 0.9
PECAM1	2.3 ± 0.7	0.3 ± 0.1	Receptor activity	10.6 ± 0.2	2.8 ± 0.4
CCR2	3.1 ± 0.3	1.1 ± 0.1	Receptor activity	6.6 ± 2.8	3.5 ± 1.1
DDX4	2.9 ± 1.1	0.8 ± 0.2	RNA helicase activity	4.4 ± 0.6	0.3 ± 0.2
HMOX1	6.7 ± 0.1	4.3 ± 0.3	Oxidoreductase activity	11.0 ± 4.1	2.9 ± 1.5
NDST1	3.8 ± 0.2	0.8 ± 0.1	N‐sulfotransferase	17.1 ± 0.1	4.9 ± 1.7
HYAL1	3.8 ± 0.1	0.5 ± 0.0	Hydrolase activity	2.4 ± 0.6	0.2 ± 0.1
LAPTM4B	4.8 ± 0.3	0.9 ± 0.2	Transmembrane transporter	3.4 ± 0.7	0.5 ± 0.1
NPC1	3.3 ± 0.1	1.4 ± 0.1	Receptor activity	5.4 ± 1.1	1.4 ± 0.3

Affected Genes Related to Inflammation. The up‐regulated genes were enhanced by LPS at least threefold higher than they were in the untreated control cells (column 2), and they were suppressed by LPS plus antofine cotreatment (column 3). Quantitative real‐time PCR with specific designed primers (Table 1) was performed to detect the expression level of these genes in LPS alone or LPS plus antofine cotreatment (column 5, 6). The PCR reaction was performed using a PikoReal™ 96 Real‐Time PCR System (Thermo Fisher Scientific Inc.), and the conditions were as follows: 45 cycles of 95°C for 5 sec and 60°C for 1 min. The data were analyzed using PiKoReal Software 2.0, exported into Excel for analysis using the ΔC_t_ (the number of PCR cycles to reach the threshold of the product detection) method, and normalized to *β*‐actin as an internal control. Results were presented as means ± SD.

**Figure 4:**
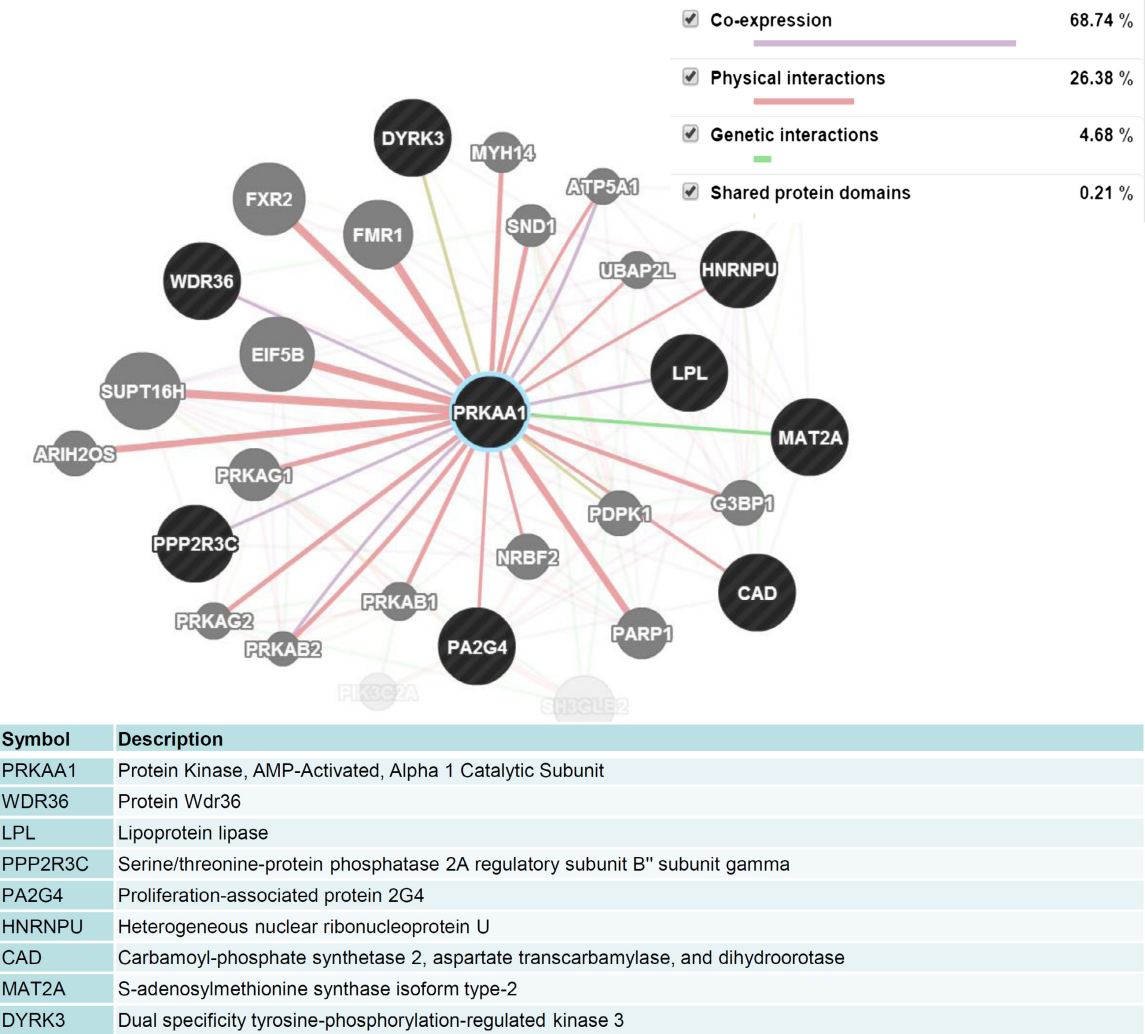
Antofine reactivated metabolism‐related genes which were suppressed by LPS stimulation. Eight of 48 genes downregulated by LPS stimulation but reactivated by antifone treatment in macrophages, namely, WDR36, LPL, PPP2R3C, PA2G4, HNRNPU, CAD, MAT2A, and DYRK3, contributed physical interactions with PRKKAA1 (AMPK), which were analyzed by software from the GeneMANIA database.

To further clarify the potential mechanism of the anti‐inflammatory effects of antofine on LPS‐induced activation of Raw264.7 murine macrophage cells, we analyzed the 48 genes that were downregulated by LPS stimulation but reactivated by antofine treatment in macrophage cells. The results, which were analyzed using the GeneMANIA database, showed that six genes, namely, WDR36, LPL, PPP2R3C, PA2G4, HNRNPU, and CAD, contributed physical interactions with PRKKAA1 (AMPK) (Fig. 4). In addition, the results indicated that MAT2A has a genetic interaction with AMPK, whereas DYRK3 shares a protein domain with AMPK. These results further indicate that AMPK may play an important role in suppressing LPS‐induced inflammation in Raw264.7 macrophage cells.

**Figure 5:**
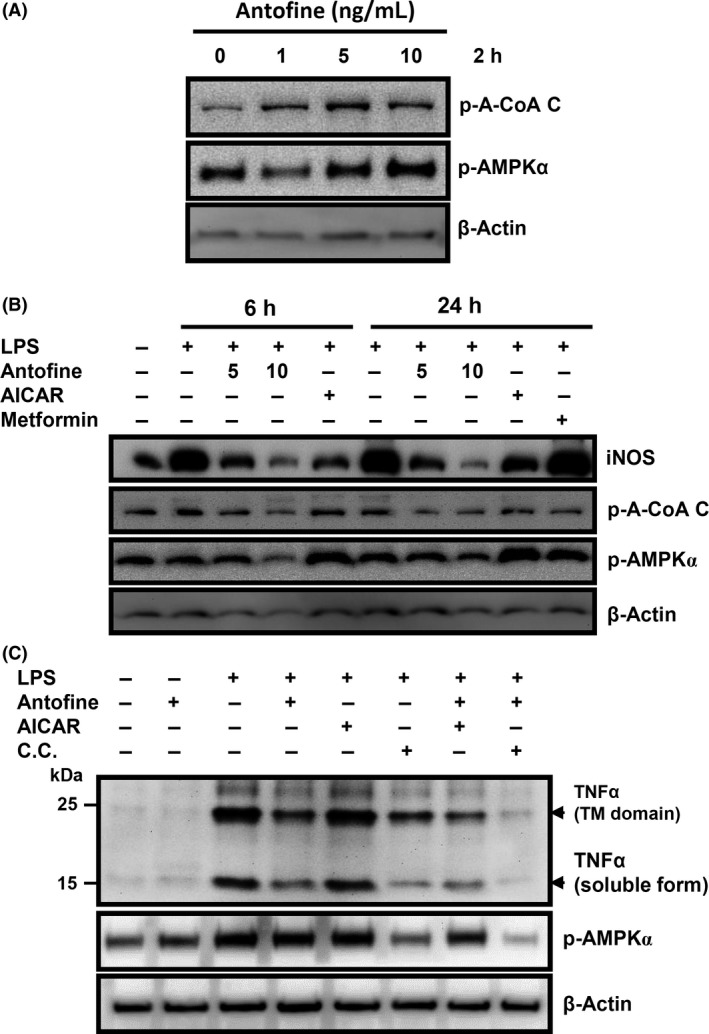
Antofine activated AMPK and suppressed LPS‐induced AMPK activation and inflammation mediators. (A) Raw264.7 macrophage cells were treated with antofine at the indicated concentrations for 2 h. The phosphorylation of acetyl‐CoA carboxylase and AMPK was examined by immunoblot. (B) Raw264.7 macrophage cells were pretreated with various compounds including antofine (5 and 10 ng/mL), AICAR (0.5 mmol/L) and Metformin (1 mmol/L) for 30 min and then stimulated by LPS (1 *μ*g/mL) for 6 h and 24 h. The production of iNOS and the phosphorylation of acetyl‐CoA carboxylase and AMPK were examined by immunoblot. (C) Raw264.7 macrophage cells were pretreated with various compounds as indicated such as antofine (10 ng/mL), AICAR (0.5 mmol/L) and Compound C (10 *μ*mol/L) for 30 min and then stimulated by LPS (1 *μ*g/mL) for 6 h. The production of TNF *α* as well as the phosphorylation of acetyl‐CoA carboxylase and AMPK was examined by immunoblot. The expression of *β*‐actin served as an internal control.

To confirm whether or not AMPK was involved in the modulation of LPS‐induced inflammation by antofine, we assayed the AMPK signaling pathway by Western blot with specific antibodies. The results showed that the AMKP*α* was activated by antofine and an AMKP*α* downstream targeted protein, acetyl‐CoA carboxylase, was significantly dose‐dependently inactivated (with phosphorylation leading to the inactivation of this enzyme) by antofine (Fig. 5A). Conversely, antofine dose dependently suppressed the production of iNOS and the phosphorylation of AMPK, both induced by LPS at the 6‐h and 24‐h time points (Fig. 5B). The AMPK activator, AICAR, was able to activate AMKP*α* but also suppressed the production of iNOS in LPS‐treated macrophage cells. Interestingly, another AMPK activator, metformin, does not suppress the production of iNOS in LPS‐treated macrophage cells. In Figure 5C, the results showed that the production of TNF*α* was associated with the activation of AMKP*α*. Antofine can suppress AMPK activities enhanced by AICAR and suppress the production of TNF*α*. However, the cotreatment of antofine and Compound C, AMPK inhibitor, can synergistically suppress the activation of AMKP*α* as well as the production of TNF*α*. It indicated that antofine inhibited the production of TNF*α* via suppressing the activation of AMPK induced by LPS stimulation.

**Figure 6:**
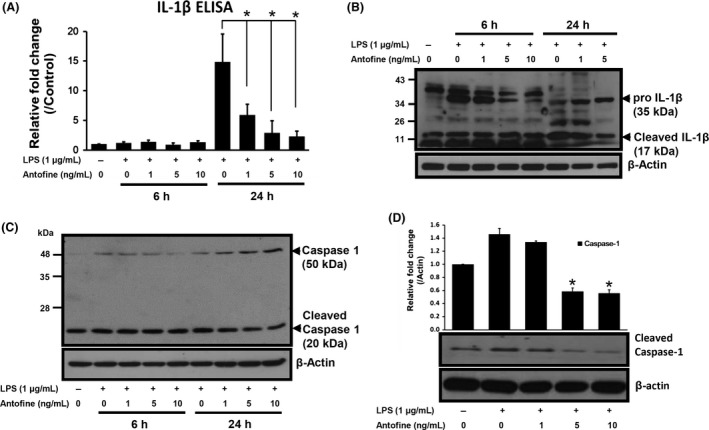
Antofine can suppress LPS‐induced caspase‐1 activation in Raw264.7 macrophage. Raw264.7 macrophage cells were pretreated with antofine at the indicated concentrations for 30 min and then stimulated by LPS (1 *μ*g/mL) for 24 h. (A) The soluble form of IL‐1*β* in cultured medium was measured by ELISA method. (B) The production of IL‐1*β* in treated cells was measured by immunoblot. The 35 kDa protein as indicated was the full length of pro‐IL‐1*β* and the 17 kDa fragment protein as indicated was the soluble form of IL‐1*β*. (C) The production of caspase‐1 in treated cells was measured by immunoblot. The 50 kDa protein as indicated was the full length of procaspase‐1 and the 20 kDa fragment protein as indicated was the active form of caspase‐1 cleaved from procaspase‐1. (D) Human bronchial epithelial cells (BEAS‐2B) were pretreated with antofine at the indicated concentrations for 30 min and then stimulated by LPS (1 *μ*g/mL) for 24 h. The production of procaspase‐1 in treated cells was measured by immunoblot. The expression of *β*‐actin served as an internal control. *P <* *0.05 versus LPS alone stimulation.

**Figure 7:**
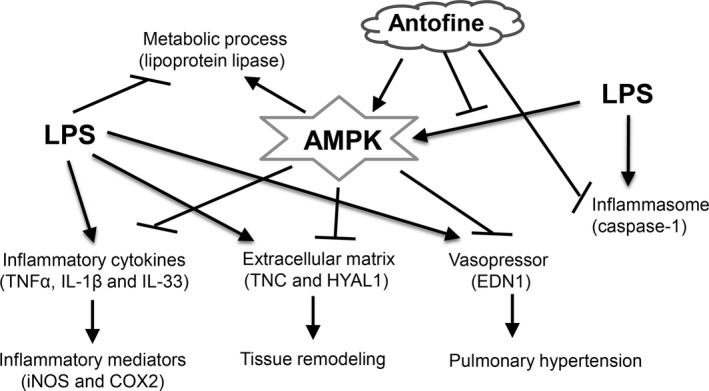
Antofine may suppress LPS‐induced inflammatory response and modulate metabolic process‐related genes via AMPK. Schematic representation of the role of AMPK as a regulator of LPS‐induced signaling pathways including proinflammatory cytokines (TNF *α*, IL‐1*β*, and IL‐33), extracellular matrix (TNC and HYAL1), vasopressor (EDN1) and inflammasome (caspase‐1) related genes.

The secretion of IL‐1*β* was low, it exhibited no significant changes at 6 h but was dramatically induced at 24 h (when compared with the LPS‐unstimulated cells) and dose dependently decreased when antofine was coincubated at the 24‐h time point (Fig. 6A). The Western blot results showed that the production of pro‐IL‐1*β* was enhanced by LPS stimulation and dose dependently suppressed by antofine treatment at the 6‐h time point. However, the soluble form of IL‐1*β* was absent in the culture medium due to un‐digested protein from pro‐IL‐1*β* at the 6‐h time point. Interestingly, the protein of pro‐IL‐1*β* was dramatically digested and the soluble form of IL‐1*β* were increased in LPS‐stimulated cells at 24 h, but the digestive effect of pro‐IL1*β* was dose dependently inhibited by antofine treatment (Fig. 6B). The protein of caspase‐1, the key regulator in inflammasome mediating IL‐1*β* maturation, was in‐activated (decrease cleaved form of caspase‐1) when the cells were cotreated with antofine in a dose‐dependent manner (Fig. 6C). A similar phenomenon to that which occurred in the Raw264.7 macrophage cells was also observed in the human lung bronchial epithelial cells (Fig. 6D). These results indicated that antofine inhibits the activity of caspase‐1 and the maturation of proinflammatory cytokines such as IL‐1*β*. In conclusion, antofine may suppress LPS‐induced inflammatory responses and modulate metabolic process‐related genes via AMPK, as shown in Figure 7, which is a schematic representation of the role of AMPK as a regulator of LPS‐induced signaling pathways.

## Discussion

In this study, we demonstrated that antofine significantly suppressed the secretion of proinflammatory cytokines such as TNF*α* and IL‐1*β* and the production of iNOS in LPS‐activated Raw264.7 cells. In addition, cDNA array analysis and subsequent experiments confirmed that several inflammation‐related genes (such as ARG‐1, IL1F9, IL‐10, and IL‐33) and extracellular matrix genes (such as TNC and HYAL1), as well as a vasopressor gene (EDN1), were induced by LPS stimulation and dose dependently downregulated by antofine treatment. We also comprehensively analyzed the profiles of gene expression in Raw264.7 murine cells treated with LPS alone or cotreated with LPS and antofine using GeneMANIA software, and found that antofine not only contributes anti‐inflammatory activity but also modulates the metabolism via AMPK. Furthermore, we also found that antofine modulates the activity of caspase‐1, the key regulator in inflammasome mediating IL‐1*β* maturation, in Raw264.7 cells. These data indicated that antofine potentially can not only contribute an anti‐inflammatory effect but can also attenuate the metabolic disorders induced by inflammation via AMPK.

Our data showed that antofine can modulate the expression of lung fibrosis‐related cytokines such as IL‐10 and IL‐33. IL‐33, a member of the IL‐1 family of cytokines that signals through the ST2 receptor, has recently been identified as a key regulator of inflammatory and immune processes. IL‐33 expression has been found to be increased in several pathological conditions, such as in the lung epithelial cells of asthmatic patients (Prefontaine et al. 2010) and COPD patients (Byers et al. 2013). In addition, several recent studies have shown that elevated IL‐33 also promotes the initiation and progression of lung fibrosis (Luzina et al. 2013; Li et al. 2014). IL‐10 is known as an anti‐inflammatory cytokine involved in the processing of immunosuppressive effects which are necessary for regulating inflammation. Sun et al. (2011) have demonstrated that IL‐10 drives fibrocyte recruitment to the lung and promotes the development of lung fibrosis. Intriguingly, we found that IL‐33 and IL‐10 were both elevated by LPS stimulation and then suppressed by antofine treatment in macrophage cells. It seems that antofine can potentially inhibit lung inflammation as well as any subsequent lung fibrosis through the suppression of these profibrogenic cytokines induced by LPS stimulation. Furthermore, we also found that LPS‐induced expressions of extracellular matrix genes such as TNC and HYAL1, as well as a vasopressor gene, EDN1, all of which are involved in the process of lung fibrosis, were also dose dependently downregulated by antofine treatment. Cumulative data suggest that antofine can not only suppress endotoxin‐induced inflammation in macrophage cells but can also prevent subsequent tissue fibrosis through the downregulation of profibrogenic cytokines and related fibrogenic genes. However, this interesting hypothesis should be addressed in the future in animal models.

This study also showed that antofine can modulate the secretion of several proinflammatory cytokines such as IL‐1*β* and TNF*α*, although it does not modulate the secretion of IL‐6 (data not shown). Our data showed that antofine dose dependently suppressed the production and secretion of TNF*α* in the early phase of inflammation when exposed to LPS. The soluble form of IL‐1*β* was digested from pro‐IL‐1*β* by caspase‐1, which was activated in the late phase of inflammation. Therefore, this can explain why the secretion of IL‐1*β* in the culture medium was low at 6 h but dramatically enhanced at 24 h when compared with the LPS‐unstimulated cells (Fig. 6A). However, these results implied that antofine may modulate LPS‐induced inflammation in macrophage cells via an intracellular signaling network. The results of cDNA microarray analysis further showed that antofine, under the optional window dosage (~10 ng/mL), can significantly restore the downregulated genes suppressed by LPS stimulation, a majority of which (63%, 48 of 76) are involved in the metabolic process. Interestingly, the results further indicated that PRKKAA1 (AMPK) can interact with most of these metabolism‐related genes restored by antofine (Fig. 4), and this phenomenon indicates that AMPK signaling may play an important role in the suppression of LPS‐induced inflammation by antofine. Numerous studies have also reported that the activation of AMPK signaling downregulates the function of the NF‐kB system via its downstream mediators, for example SIRT1, the FoxO family (Zhang et al. 2010; Salminen et al. 2011; Huang et al. 2015). Yang et al. (2010) revealed that the activation of *α*1AMPK can suppress NF‐kB signaling and fatty acid‐induced inflammation; conversely, the suppression of *α*1AMPK reverse this inhibition. Activation of AMPK by resveratrol inhibits LPS‐induced activation of NF‐*κ*B and the expression of COX2 in macrophage cells (Yi et al. 2011). Interestingly, we found that antofine alone can activate AMPK activity, but it also suppresses the LPS‐induced activation of AMPK in macrophage cells. However, the detailed mechanism of antofine regarding its counteracting of the activation of AMPK induced by LPS stimulation remains unknown. In addition, Jang et al. (2014) demonstrated that antofine can contribute antiadipogenic activity at low concentrations (0.01–10 nmol/L) via the suppression of the PPAR*γ* gene in adipocytes. Our current data also showed that antofine can restore the expression of lipoprotein lipase (LPL) suppressed by LPS‐induced inflammation. This finding indicated that antofine may be a promising candidate for controlling obesity and metabolic disorders.

Activation of inflammasome is involved in obesity‐related diseases, such as type II diabetes, and in cardiovascular diseases (Martinon et al. 2009; Horng and Hotamisligil 2011; Wen et al. 2011). AMPK regulates inflammasomal activation by inhibiting NF‐KB activation (Hattori et al. 2008). We also confirmed that antofine can suppress the activity of caspase‐1 in Raw264.7 cells when cotreated with LPS. This means that antofine can modulate the activity of inflammasomes that are activated in obesity‐related diseases. The data further indicated that antofine potentially can not only contribute an anti‐inflammatory effect but can also attenuate the metabolic disorders induced by chronic inflammation via AMPK. However, the details of the mechanism by which this occurs will have to be determined by further research conducted with other animal models.

## Author Contribution

Shao‐Ting Chou, Hwei‐Ling Chou, and Jau‐Chen Lin participated in research design. Fang Jung and Shih‐Hsing Yang conducted the experiments. Fang Jung, Shih‐Hsing Yang, and Jau‐Chen Lin performed data analysis. Guey‐Mei Jow and Jau‐Chen Lin wrote or contributed to the writing of the manuscript.

## Disclosure

None declared.
